# Characterization of a mycovirus associated with the brown discoloration of edible mushroom, *Flammulina velutipes*

**DOI:** 10.1186/1743-422X-7-342

**Published:** 2010-11-25

**Authors:** Yumi Magae, Masahide Sunagawa

**Affiliations:** 1 Department of Applied Microbiology, Forestry and Forest Products Research Institute, Tsukuba, Ibaraki 305-8687, Japan

## Abstract

**Background:**

A mycovirus previously identified in brown discolored fruiting bodies of the cultivated mushroom *Flammulina velutipes *was characterized. We tentatively named the virus the *F. velutipes *browning virus (FvBV).

**Results:**

Purified FvBV particles contained two dsRNA genomes (dsRNA1 and 2). The complete sequence of dsRNA1 was 1,915 bp long, containing a single open reading frame (ORF) that encoded 580 amino acids of a putative 66-kDa RNA-dependent RNA polymerase (RdRp). dsRNA2 was 1,730 bp long containing a single ORF encoding 541 amino acids of a putative 60-kDa coat protein (CP1). Phylogenetic analysis of the RdRp sequences revealed FvBV to be a *Partitivirus*, most closely related to *Chondrostereum purpureum *cryptic virus. An RT-PCR assay was developed for the amplification of a 495-bp cDNA fragment from dsRNA encoding the CP1. When wild *F. velutipes *isolated from various parts of Japan were examined by RT-PCR assay, three isolates from the central region of Japan contained FvBV. One wild strain infected with FvBV was isolated in Nagano prefecture, where brown discoloration of white cultivated strains has occurred. Fruiting bodies produced by virus-harboring and virus-free *F. velutipes *were compared.

**Conclusions:**

Cap color of the fruiting bodies of *F. velutipes *that contained *Partitivirus *FvBV was darker than FvBV-free fruiting bodies. The use of RT-PCR enabled association of FvBV and dark brown color of the fruiting body produced by *F. velutipes *strains.

## Background

At the time mycoviruses were discovered in the white button mushroom, *Agaricus bisporus*, in 1962 [1], *Lentinula edodes *was the only artificially cultivated mushroom in Japan. Since then, the number of cultivated mushroom species has greatly increased: *L. edodes*, *Flammulina velutipes*, *Hypsizygus marmoreus*, *Pholiota nameko*, *Grifola frondosa*, and *Pleurotus eryngii *are cultivated daily. As the mushroom industry in Japan continues to grow, various abnormal symptoms during cultivation have been observed. Symptom that resembled abiotic disorders of *A. bisporus *[2] first became apparent with cultivated strains of *F. velutipes *[3,4]. One of the abnormal symptoms was the spontaneous appearance of brown discolored fruiting bodies among the white ones. We detected spherical virus particles in the brown discolored fruiting bodies of two different cultivated *F. velutipes *[3,5]. We tentatively named the virus FvBV, for *Flammulina velutipes *browning virus.

Browning is also a typical symptom observed in MVX (Mushroom virus X) disease in *A. bisporus *[6-8]. With MVX, complex patterns of more than 26 dsRNA bands are detected by agarose gel electrophoresis. Four low molecular weight dsRNAs have been specifically linked with the browning syndrome [6] together with some bacterial agent [8].

In the present study, we determined the sequences of the dsRNA genomes of FvBV. The two dsRNA isolated from the purified virus particles potentially coded for RNA-dependent RNA polymerase (RdRp) and a coat protein. Based on the phylogenetic analysis of the RdRp sequence, FvBV belongs to the family *Partitiviridae*. From the nucleotide sequence of the FvBV coat protein, RT-PCR primers were designed. To estimate the influence of FvBV on fruiting body color, fruiting bodies produced by virus-harboring strains and virus-free strains of *F. velutipes *were compared. Based on the RT-PCR assay developed in this study, FvBV and the dark brown cap color of the fruiting bodies were clearly linked.

## Results

### Molecular characterization of *FvBV*

**Figure 1:**
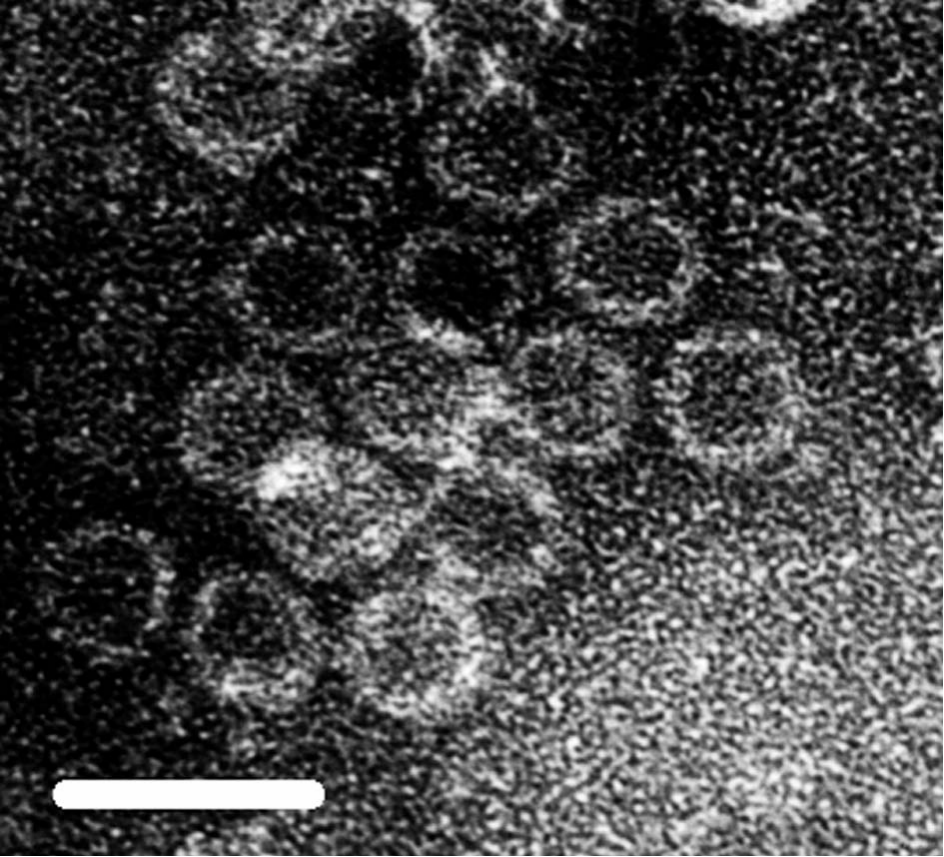
**Electron micrograph of purified FvBV virions**. The bar represents 50 nm.

The complete nucleotide sequences of the two dsRNAs isolated from the purified virions (Figure 1) were determined. The sequences are deposited in DDBJ under the accession numbers of AB465308 and AB465309.

**Figure 2:**
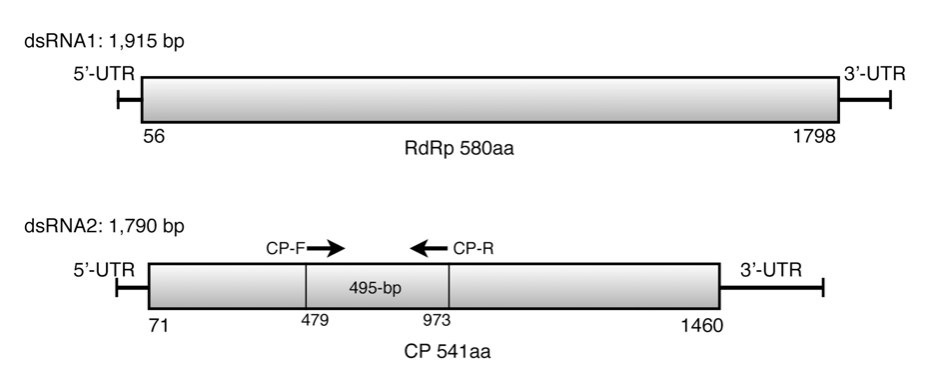
**Diagrammatic representation of genome organization of FvBV**. dsRNA1 encodes putative RdRp and dsRNA2 encodes putative coat protein. Primers CP-F (5'-GCCCGCATCATTACTGTTCT-3') and CP-R (5'-TATCGGTATCAGGGCCGTAG-3') were used for RT-PCR detection of FvBV.

dsRNA1: The sequence of dsRNA1 was 1,915 bp in length, containing a single open reading frame (ORF) starting at position nt 56 and ending at nt 1798, which encoded a putative protein of 580 amino acids with a deduced molecular mass of 66,716 Da (Figure 2). A PSI-BLAST [9] search of the deduced amino acid sequence of dsRNA1, revealed the presence of the conserved motif (pFam cd01699 RNA_dep_RNAP, 3e^-6)^) [10] for RdRp within the ORF. RdRp is an essential protein encoded in the genomes of all RNA containing viruses with no DNA stage.

**Figure 3:**
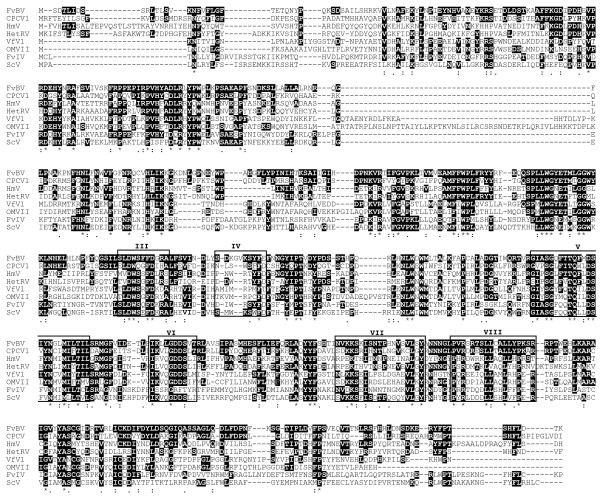
**Alignment of RdRp sequences**. The six conserved motifs (III~VIII) of RdRp are located. Identical residues are in black background. : and . indicate similar residues. FvBV: *F. velutipes *browning virus, CpCV1: *C. purpureum *cryptic virus1, HmV: *H. mompa *dsRNA mycovirus, HetRV: *Heterobasidion *RNA Virus, VfV1: *Vicia faba *partitivirus 1, OMVII: Oyster mushroom isometric virus II, FvIV: *F. velutipes *isometic virus, ScV: *Sclerotinia sclerotiorum *partitivirus S

**Figure 4:**
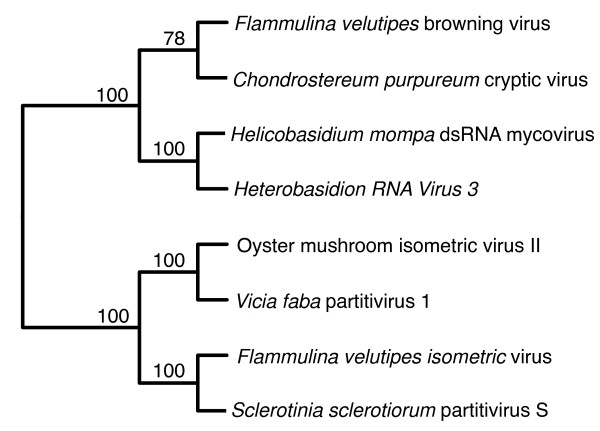
**Unrooted phylogenetic tree generated from amino acid sequences of RdRp**. The unrooted phylogenetic tree of the aligned sequences was constructed based on the neighbor-joining method with a 1,000 replicate bootstrap search. The alignment was carried out with the program L-INS-i of MAFFT and visualized on the Web server http://align.genome.jp/mafft/. Numbers at the nod indicate percentage of bootstrap supports from 1,000 replicates.

Sequence identities and E values of proteins deposited in GenBank that showed significant similarity with the putative RdRp encoded by dsRNA 1 are listed in Table 1. All of these viruses belonged to the family *Partitiviridae *[11]. Multiple alignments of RdRp amino acid sequences of the viruses listed in Table 1 were constructed using the MAFFT program [12]. Conserved motifs III-VIII of RdRp of dsRNA viruses [13] were found (Figure 3). An unrooted phylogenetic tree based on the alignment with bootstrap of 1000 was made (Figure 4). RdRp of FvBV was grouped together with a partitivirus in the basidiomycete fungus *Chondrostereum purpureum *and placed adjacent to a group containing two partitiviruses of pathogenic basidiomycete fungus: *Helicobasidium mompa *dsRNA mycovirus and *Heterobasidion RNA Virus *(Figure 4).

**Table 1:** RdRp sequences of viruses included in the phylogenetic tree

Virus	Accession no.	E-value	Identity	Similarity	References
*Flammulina velutipes *browning virus	BAH56481				This study
*Chondrostereum purpureum *cryptic virus 1	CAQ53729	7e^-146^	270/564 (48%)	363/564 (65%)	[20]
*Helicobasidium mompa *dsRNA mycovirus	BAC23065	1e^-105^	226/559 (41%)	316/559 (57%)	[21]
*Heterobasidion RNA Virus 3*	AC037245	2e^-95^	204/504 (41%)	286/504 (57%)	[22]
*Vicia faba *partitivirus 1	ABJ9996	2e^-79^	199/548 (37%)	288/548 (53%)	[23]
*Flammulina velutipes *isometic virus	BAH08700	3e^-76^	183/492 (38%)	267/492 (55%)	
*Sclerotinia sclerotiorum *partitivirus S	YP_003082248	2e^-74^	179/477 (38%)	254/477 (54%)	
Oyster mushroom isometric virus II	AAP74192	2e^-64^	183/557 (33%)	272/557 (49%)	

E-values, identity and similarity values were obtained from PSI-BLAST searches of the deduced amino acids data of RdRp sequences.

Viruses that showed bootstrap value above 70% were included in the phylogenetic tree.

**Figure 5:**
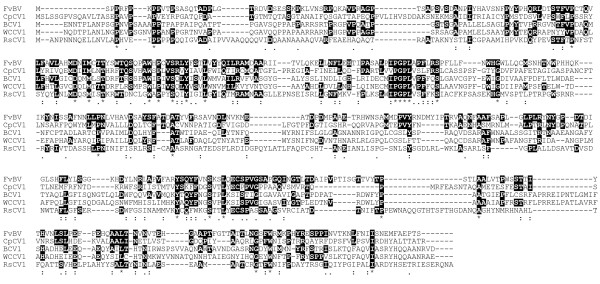
**Alignment of CP sequences**. Identical residues are in black background. : and . indicate similar residues. FvBV:*F. velutipes *browning virus, CpCV1:*C. purpureum *cryptic virus1, BcV1:Beet cryptic virus 1, WCCV1: White clover cryptic virus 1, RsCV1:*R. sativus *cryptic virus 1

dsRNA2: The sequence was 1,730 bp, with a single ORF starting at nt 71 and ending at 1,460 (Figure 2). The deduced amino acid sequence coded for putative protein of 541 amino acids with a molecular mass of 60,513 Da. Sequence identities and E values of proteins deposited in GenBank that showed significant similarity with the putative coat protein encoded by dsRNA 2 are listed in Table 2. Multiple alignments of coat protein amino acid sequences of the viruses listed in Table 2 are shown (Figure 5).

**Table 2:** Coat protein sequences of viruses that showed high similarity with CP1 of FvBV

Virus	Accession no.	E-value	Identity	Similarity	References
*Flammulina velutipes *browning virus	BAH56481				This study
*Chondrostereum purpureum *cryptic viru 1	CAQ53730	1e^-19^	102/372 (28%)	158/372 (43%)	[20]
Beet cryptic virus 1	YP_002308575	1e^-13^	118/462 (26%)	182/462 (40%)	[24]
Whit clover cryptic virus 1	YP_086755	1e^-13^	120/496 (25%)	185/496 (38%)	[25]
*Raphanus sativus *cryptic virus 1	ABX79673	2e^-13^	102/421 (25%)	174/421 (42%)	

E-values, identity and similarity values were obtained from PSI-BLAST searches of the deduced amino acids data of coat or capsid protein sequences.

### Fruiting body production and RT-PCR analysis

**Figure 6:**
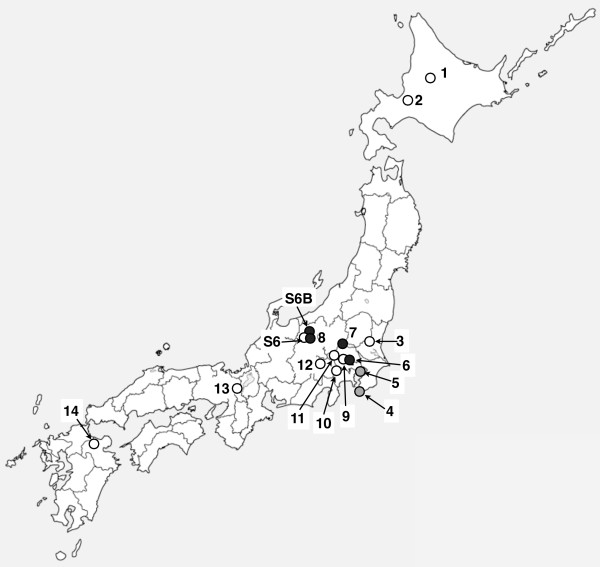
**Location in Japan for isolation of each strain**. Each number designates the No. of isolate. White circle; dsRNA(-), grey circle; dsRNA (+), black circle; isolate containing FvBV.

**Figure 7:**
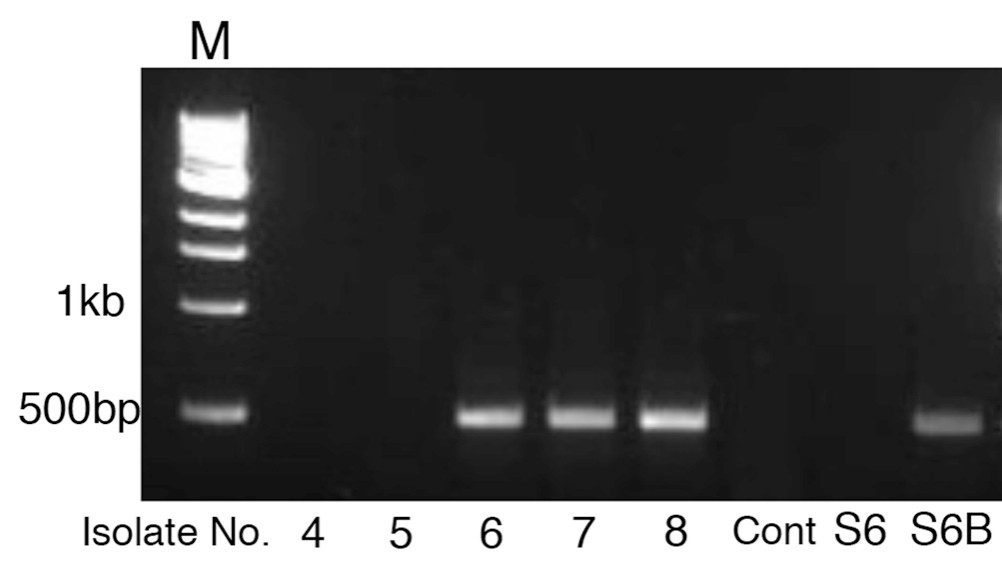
**Results of RT-PCR assay that amplified the cDNA encoding the coat protein of FvBV**. From left to right, template used for RT-PCR; dsRNA isolated from Isolate No. 4, 5, 6, 7, 8, TE (Control), and cultivated strain S6 and S6B.

**Figure 8:**
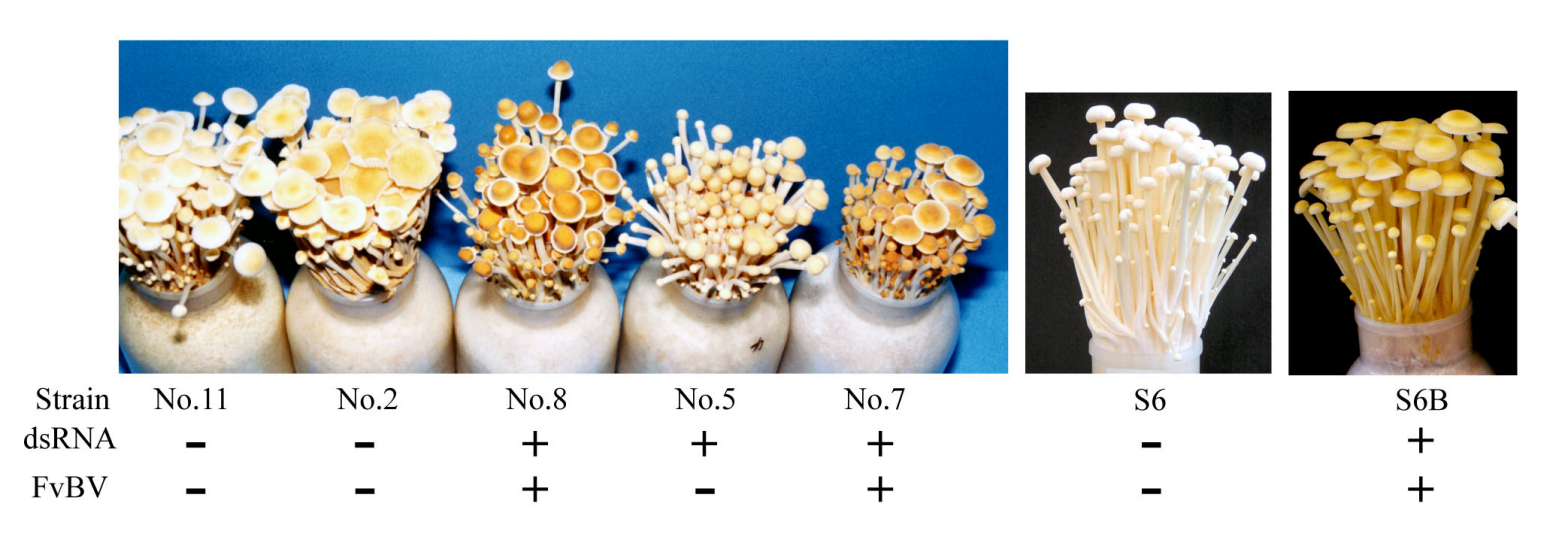
**Fruiting body production of *F. velutipes***. From left to right; Fruiting bodies of virus-free (isolate No. 11, 2 and S6) and virus-containing (isolate No. 8, 5, 7 and S6B) *F. velutipes*. The result shows that all of the *F. velutipes *containing FvBV (Isolate No.7, 8 and S6B) produced fruiting bodies with dark brown caps.

Among the 14 wild isolates (Table 3) examined for dsRNA, five from the central region of Japan contained dsRNA (Figure 6). Based on the RT-PCR analysis, three of the five isolates contained FvBV (Figure 7). Among the five dsRNA-harboring wild isolates, isolate No. 4 did not produce fruiting bodies. Also, Isolate No. 6 did not produce enough fruiting bodies (yield 37 g) to make an accurate comparison that it was eliminated from the test. Other dsRNA-harboring wild isolates, including isolate 5 (yield 107 g), 7 (yield 62 g) and 8 (yield 100 g) produced enough fruiting bodies for color comparison. As shown in the photograph (Figure 8), dsRNA-free wild isolate No. 2 (yield 104 g) and No. 11 (yield 98 g) produced fruiting bodies with paler-colored caps while fruiting bodies of the two isolates (No.7 and 8) containing FvBV were dark brown (Figure 8). Color of fruiting bodies produced by isolate No.5, which contained dsRNA but not FvBV, was much paler compared to isolate 7 and 8. All the *F. velutipes *harboring FvBV (two wild and one cultivated) produced dark brown fruiting bodies.

**Table 3:** List of Flammulina velutipes isolates

Isolate No.	Location	Isolated Year	dsRNA
1	Furano, Hokkaido	1984	-
2	Sapporo, Hokkaido	1984	-
3	Kukizaki, Ibaraki	1984	-
4	Tateyama, Chiba	1983	+
5	Kimitsu, Chiba	1983	+
6	Komaba, Tokyo	1984	+
7	Hidaka, Saitama	1984	+
8	Sanada, Nagano	1987	+
9	Meguro, Tokyo	1950	-
10	Takao Mt., Tokyo	1985	-
11	Tanzawa Mt., Kanagawa	1979	-
12	Sudama, Yamanashi	1987	-
13	Daimonji, Kyoto	1954	-
14	Kumato Mt., Ohita	1979	-
S6	Matsushiro, Nagano	1997	-
S6B	Matsushiro, Nagano	1997	+

## Discussion

In our previous study, we investigated the presence of dsRNA elements in cultivated strains of *F. velutipes *to determine whether viruses are involved in the malformation of fruiting bodies. All of these strains were, however, dsRNA free [3]. Unexpectedly, spherical mycoviruses were detected in *F. velutipes *that showed brown discoloration during cultivation. This finding suggested that mycovirus causes brown discoloration of the mushroom. We tentatively named the virus *Flammulina velutipes *browning virus, or FvBV. Because browning reduces the crop value of white mushrooms, FvBV was further characterized in this study.

In this study, the sequences of two dsRNAs contained in the viral particles of FvBV were determined. dsRNA1 coded for putative RdRp with a sequence showing significant homology with members of the family *Partitiviridae *(Table 1). The family *Partitiviridae *consists of fungal and plant viruses with isometric virions, 30-35 nm in diameter, with a genome of two dsRNA segments each of 1.4-2.2 kb. The viruses are associated with latent infections of fungal or plant hosts [11]. dsRNA2 coded for putative coat protein with a sequence showing high homology with partitiviruses (Table 2). But the E-values were much larger compared to RdRp sequences.

It is noted that one of the three wild isolates (Isolate No. 8) harboring FvBV was isolated in Nagano prefecture, where spontaneous discoloration of cultivated *F. velutipes *has occurred at two independent locations (Figure 6). Because the mycelium of strain S6B does not show incompatibility against strain S6 (data not shown), it is unlikely that strain S6B is a wild *F. velutipes *contaminated during cultivation. Thus, presumably, there was horizontal transfer of the virus from a wild isolate to a cultivated strain of *F. velutipes *within Nagano prefecture.

The result of mushroom cultivation (Figure 8) shows that most of the isolates harboring mycoviruses in *F. velutipes *do not influence fruiting body development. All the virus-harbouring isolates except for Isolate No.4 produced fruiting bodies. The colony of strain No.4 showed very slow growth, flat morphology and incompatibility as well as fruiting body productivity is lost. These characteristics are similar to the hypovirulence identified in pathogenic fungi [14]. dsRNA of mycovirus in this degenerate wild strain (designated as *F. velutipes *isometric virus; FvIV), has been sequenced and deposited in DDBJ under the accession no. of AB428575. FvIV is studied in detail in another study and submitted for publication.

The color of the fruiting body, as can be seen in the photograph, shows that virus-free *F. velutipes *fruiting bodies are whiter (Figure 8). Based on the results of the RT-PCR assay and fruiting body production, the presence of FvBV in mycelium and the dark brown color of fruiting bodies were clearly correlated.

In *A. bisporus*, brown discoloration of fruiting body caused by bacterial or fungal infection is known [15,16]. Toxins produced by the bacteria can induce brown coloration of *A. bisporus *[17]. A quantitative trait locus of *A. bisporus *resistance to *P. tolassi *is closely linked to natural cap color [18].

## Conclusions

Partitivirus in fungi has been associated with only symptomless infections [11]. For example, a partitivirus in oyster mushroom does not affect its host's phenotype [19]. In this respect, FvBV is possibly unique because it is associated with a specific symptom of *F. velutipe*s. Although Koch's postulates have not been established yet, an association between a browning of fruiting body and a partitivirus in *F. velutipes *became evident in this study.

## Methods

### Strains

Viral particles were purified from mycelium of *F. velutipes *strain S6B. Strain S6B is a variant of the cultivated *F. velutipes *strain Shinano No. 6 (designated as S6) that contained dsRNAs and showed brown discoloration of the fruiting bodies [5]. The 14 wild isolates and 2 cultivated strains used in this study are listed in Table 3.

### Culturing

The strains were routinely maintained on MA (2% malt extract, 1.8% agarose). Cultures for extraction of dsRNA were grown in 300-ml Erlenmeyer flasks containing 150 ml of GYM (1% glucose, 0.4% yeast extract, and 1% malt extract) at 24°C, stationary for 3 to 4 weeks.

### dsRNA extraction

Viral particles of *F. velutipes *were precipitated with PEG 8000 and NaCl as described previously [3]. Total RNA from the PEG precipitate suspended in TES (10 mM Tris-HCl, 1 mM EDTA, 0.15 M NaCl, pH 7.0) was isolated using the QIAmp Viral RNA Mini Kit (Qiagen, Chatsworth, CA, USA) according to the manufacturer's instructions. dsRNA for agarose gel electrophoresis was prepared from the viral RNA by DNase I (Promega, Madison, WI, USA) and S1 nuclease (Takara Bio, Ohtsu, Japan) digestion. The nuclease-digested product was precipitated with isopropanol after extraction with Sepasol-RNA I Super (Nacalai Tesque, Kyoto, Japan). Total dsRNA was electrophoresed on agarose gel, stained with ethidium bromide, and observed under UV illumination.

### Purification of viral particles

The PEG precipitate of viral particles was subjected to 30-40% sucrose density-gradient centrifugation for 2 h at 100,000 × g at 4°C. After the ultracentrifugation, the sample was fractionated into 2-ml volumes, and 100 μl of each fraction was assessed for the presence of dsRNA using the QIAmp Viral RNA Mini Kit (Qiagen). Fractions containing the dsRNA were collected, and viral particles were confirmed by electron microscope observation using a transmission electron microscope (H-7000; Hitachi, Tokyo) after negative staining with 2% aqueous uranyl acetate. dsRNA was extracted from the purified viral particles using the RNeasy Mini Elute Clean-up Kit (Qiagen).

### dsRNA sequence

dsRNA extracted from the purified virions was cloned using the Takara small RNA Cloning Kit (Takara Bio) according to the manufacturer's instructions, with a slight modification: RevaTraAce (Toyobo, Osaka, Japan) was used for cDNA synthesis instead of the M-MLV RTase included in the kit, with the aim of generating a longer cDNA. The cDNA was cloned into the pGEM-T easy vector (Promega, Madison, WI, USA) according to the manufacturer's instructions and transformed into *E. coli *DH5α (Toyobo). Cloned DNA was PCR amplified using Insert Check Ready (Toyobo). The DNA was sequenced using the BigDye Terminators v1.1 Cycle Sequencing Kit (Applied Biosystems, Foster City, CA, USA) with an ABI Prism 3100 Genetic Analyzer (Applied Biosystems).

### Sequence analysis

A PSI-BLAST search [9] was performed with the predicted amino acid sequences of the cDNA generated from dsRNA1 and 2. Sequence alignment was performed using the MAFFT program L9INS-I (available at http://align.bmr.kyushu-u.ac.jp/mafft/online/server/index.html) [12]. Neighbour joining with 1,000-boot strapping was performed and phylogenetic tree was constructed with sequences of bootstrap value above 70%. For visually constructing phylogenetic tree, the MAFFT program on the Web server http://align.genome.jp/mafft/ was used. Sequences used for constructing the phylogenetic tree are summarized in Table 1.

### RT-PCR

Primers for RT-PCR were designed from the sequence of the putative CP1. Primers CP-F (5'-GCCCGCATCATTACTGTTCT-3') and CP-R (5'-TATCGGTATCAGGGCCGTAG-3') were designed for the amplification of the 495-bp product from nt 479 to 973 of the CP1 DNA using Primer version 3.0 (Whitehead Institute for Biomedical Science). RT-PCR was performed with PEG-precipitated dsRNA prepared as described above with 20 pmol of both primers and RT-PCR quick Master Mix (Toyobo). The dsRNA was denatured at 100°C for 10 min. and immediately cooled on ice. Reverse transcription was performed as follows: 90°C for 30 sec, 60°C for 30 min, and 94°C for 1 min. The following PCR amplification was 40 cycles at 94°C for 30 sec, 55°C for 30 sec, 72°C for 1 min with a final extension of 72°C for 7 min. PCR products were electrophoresed on agarose gels, stained with ethidium bromide, and observed under UV illumination.

### Fruiting body production

Fruiting bodies were produced using sawdust supplemented with rice bran as the substrate, contained in polypropylene bottles of 800 ml as described in [4].

## Competing interests

The authors declare that they have no competing interests.

## Authors' contributions

YM purified the virus, isolated dsRNA, did the phylogenetic analysis and wrote the manuscript. MS cloned the sequences, designed RT-PCR primers and performed the RT-PCR analysis. All authors read and approved the final manuscript.
